# Identification of a regulation network in response to cadmium toxicity using blood clam *Tegillarca granosa* as model

**DOI:** 10.1038/srep35704

**Published:** 2016-10-20

**Authors:** Yongbo Bao, Xiao Liu, Weiwei Zhang, Jianping Cao, Wei Li, Chenghua Li, Zhihua Lin

**Affiliations:** 1 Zhejiang Key Laboratory of Aquatic Germplasm Resources, College of Biological & Environmental Sciences, Zhejiang Wanli University, Ningbo, Zhejiang, 315100, P.R. China; 2 Department of Systems biology, GFK, Shanghai Biotech Inc., Shanghai, 201112, P.R. China; 3 School of Marine Scienes, Ningbo University, Ningbo, Zhejiang, 315010, P.R. China; 4 Ningbo Yinzhou Measurement and Test Center for Quality and Technique Supervising, Ningbo, Zhejiang, 315100, P.R. China

## Abstract

Clam, a filter-feeding lamellibranch mollusk, is capable to accumulate high levels of trace metals and has therefore become a model for investigation the mechanism of heavy metal toxification. In this study, the effects of cadmium were characterized in the gills of *Tegillarca granosa* during a 96-hour exposure course using integrated metabolomic and proteomic approaches. Neurotoxicity and disturbances in energy metabolism were implicated according to the metabolic responses after Cd exposure, and eventually affected the osmotic function of gill tissue. Proteomic analysis showed that oxidative stress, calcium-binding and sulfur-compound metabolism proteins were key factors responding to Cd challenge. A knowledge-based network regulation model was constructed with both metabolic and proteomic data. The model suggests that Cd stimulation mainly inhibits a core regulation network that is associated with histone function, ribosome processing and tight junctions, with the hub proteins actin, gamma 1 and Calmodulin 1. Moreover, myosin complex inhibition causes abnormal tight junctions and is linked to the irregular synthesis of amino acids. For the first time, this study provides insight into the proteomic and metabolomic changes caused by Cd in the blood clam *T*. *granosa* and suggests a potential toxicological pathway for Cd.

One of the most important concern in marine and coastal environments is the heavy metal due to their toxic effects on marine organisms^1^,^2^. Among them, cadmium (Cd), a non-essential metal element required for many of living organisms, can easily enter the environment as a pollutant from many anthropogenic activities^3^. The development and industrialization of coastal zones has contributed greatly to Cd pollution in marine environments^4^. As a result, Cd is one of seven most common released heavy metals for the environment (Cd, Cr, Cu, Hg, Ni, Pb, and Zn)^5^ and affects many invertebrates in marine, such as gastropod mollusks and bivalve^6^. In the East China Sea, especially along the coasts in Zhejiang Province, Cd has been determined as the most abundant heavy-metal pollutant in *Tegillarca granosa*^7^. Therefore, bivalve responses to Cd contamination represent an important research topic in environmental toxicology, public health and costal conservation^8^.

Molluscs are known and used worldwide as healthy indicators of oceans, because of their ability of metal concentration accumulation and integration^9^. Despite many studies on Cd-induced toxicological effects at the physiological level, the mechanisms underlying the observed responses are still poorly understood. Previous studies have found some proteins, such as metallothionein (MT), as bioindicators of response to induced Cd exposure^10^,^11^,^12^ or superoxide dismutase (SOD) activity as an index to estimate Cd induction level^13^. However, change in MT or SOD level is only one of many underlying mechanisms related to Cd resistance in animals.

Metabolomics is a high-throughputs technology for detecting small metabolites and molecular products of metabolism^14^,^15^. ^1^H NMR spectroscopy is often used to detect a large range of endogenous metabolites in an organism. Rich quantitative and structural information by ^1^H NMR allows analyzing many metabolites simultaneously. However, reports of the application of NMR-based toxicology studies in marine invertebrates have been limited to date^16^,^17^,^18^,^19^. High applicability and reliability of this technology has been demonstrated by applying it to investigate the toxicological mechanisms of cadmium and copper on green mussels^20^. Blaise *et al*.^21^ applied ^1^H NMR to reveal the latent phenotype associated to superoxide dismutase and catalase *Caenorhabditis elegans* mutations, and other studies also used ^1^H NMR to obtain metabolic profiles of *C*. *elegans* responses to cadmium^22^.

Proteomics is a large-scale technology in study of protein structure and function. It has shown its potential to enhance understanding of stress response mechanisms at molecular level^23^. iTRAQ, isobaric tags for relative and absolute quantification, uses labeled peptides for protein identification and quantification. Because the labeled peptides can be identified by sensitive mass spectrometers, it makes iTRAQ the one of currently widely used proteomic approach^24^. A number of studies have characterized the metabolomics or proteomic responses to several stressors in mollusks, including heavy metal exposure, arsenic exposure, and pathogen infection^3^,^25^,^26^,^27^,^28^. For instance, Stress responses in the gills of the pacific oyster *Crassostrea gigas* were conducted by iTRAQ proteomic analysis^28^. iTRAQ-based proteomics also revealed novel proteins involved in pathogen challenge in Sea Cucumber *Apostichopus japonicas*^8^. However, to our knowledge, no iTRAQ-based proteomic and NMR-based metabolomic combination studies have been conducted to understand the responses of clams to Cd. Perturbation in metabolites and proteins that are involved in same metabolic pathways can be detected by proteomics and metabolomics. Therefore, their combination can lead to a better understanding of toxicological effects of contaminants in bioindicator species^29^,^30^. For example proteomic and metabolomic analysis revealed gender-specific responses of mussel *Mytilus galloprovincialis* to tetrabromodiphenyl ether^30^, responses of clam *R*. *philippinarum* to arsenic exposure under different salinities^31^, and responses of Pacific oyster *C*. *gigas* to elevated pCO_2_ exposure^32^.

Our work integrated iTRAQ and H NMR approach to detect biomarker response to Cd in gill and to elucidate the toxicological effects of Cd (25 μg/L 250 μg/L) in the blood of the clam *T*. *granosa* at three (24, 48 and 96 h) exposure time points. There were no obvious histopathological changes in gill at selected 250 μg/L for Cd exposures compared with the control within 96 h according to pre-experiment. Here our study aimed to address the following points: (1) Which metabolite and proteins or functional groups of proteins alter their expressions significantly in response to the three time points previously mentioned? (2) Are these responses specific or common to the three different time points? (3) Construction of a regulation network to better understand the relationship between the proteins, affected pathways and downstream metabolic compounds.

## Results and Discussion

### Cadmium bioaccumulation in the gills of blood clams

**Figure 1:**
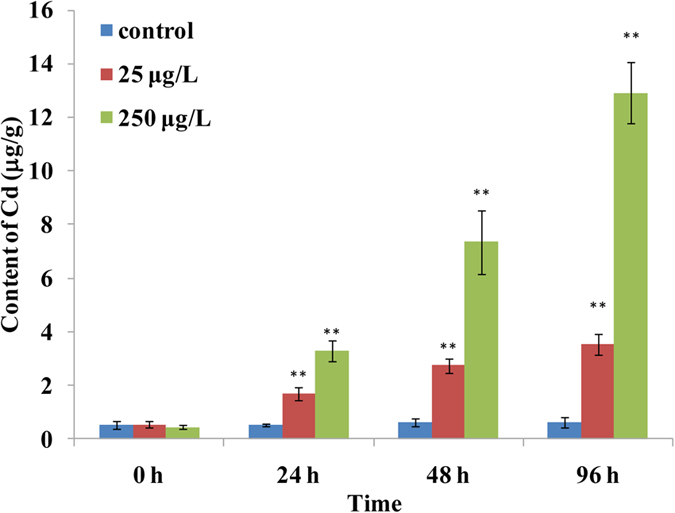
The accumulated concentrations (μg/g) of cadmium (Cd) in the gills from blood clams *Tegillarca granosa* after exposure to Cd for 24, 48 and 96 hours. The cadmium concentrations were presented as the mean ± standard deviation. The significance of the difference (**p < 0.01) between control and Cd treated-groups was tested by a one-way analysis of variance.

Figure 1 shows the accumulation of cadmium in blood clam gill tissues after 24, 48 and 96 hours of exposure. The average concentration of cadmium in gill tissues that were exposed to a low dose of cadmium (25 μg/L) was approximately 3 times higher than that of the control group after 24 hours of exposure (*p* < 0.01), while the cadmium from the high dose of cadmium (250 μg/L)-exposed group was approximately 6-fold (*p* < 0.01) higher than that of the control group. The concentrations of cadmium in blood clam gill tissue were continuously elevated with the increased exposure time for both low and high cadmium-dosed groups. The Cd concentrations in 25 and 250 μg/L cadmium-exposed blood clam samples were approximately 5 and 16 (*p* < 0.01) times higher than those of the control group after Cd exposure for 48 hours, respectively. At 96 h post exposure, the gill tissue Cd levels for the low and high Cd exposures were 6.4 and 28 times higher, respectively, than that of the control group. This finding indicates that this marine bivalve has a high accumulation of Cd and is a good bioindicator for Cd monitoring and ecotoxicology. Previous study also showed that of the five most common molluscs along Zhejiang coast in China, *T*. *granosa* had greater aptitude for bioaccumulation of Cd^7^.

### NMR-based metabolomics of Cd challenging in blood clams

Supplementary Fig. S1 displays a typical 1-D ^1^H NMR spectrum of gill tissue extracts from a control blood clam. Several different classes of metabolites were identified by the Chenomx software, including amino acids, energy storage compounds, organic osmolytes, and intermediates of the tricarboxylic acid cycle.

**Figure 2:**
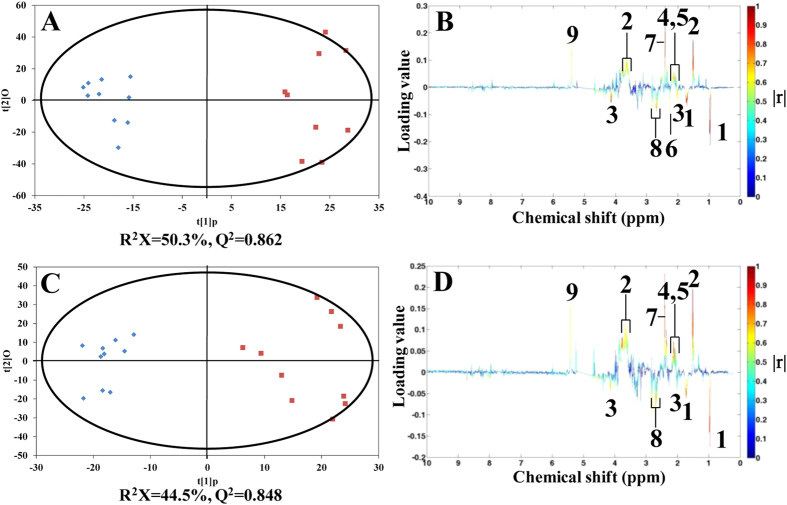
OPLS-DA score plots derived from ^1^H NMR spectra of gill tissue extracts from the control (♦) and exposed (■) blood clam groups with (**A**) 25 and (**C**) 250 μg/L Cd, respectively, after exposure for 24 hours and corresponding coefficient plots (**B**,**D**). The most significant metabolic variations between Cd exposure and control group are shown in the coefficient plots: positive directed peaks for up-regulated metabolites in Cd exposure group; negative directed peaks for down-regulated metabolites. Key: (1) leucine, (2) alanine, (3) proline, (4) glutamate, (5) glutamine, (6) acetoacetate, (7) succinate, (8) aspartate and (9) glycogen.

OPLS-DA analysis was performed on the NMR spectral data of blood clam gill tissue extracts to seek metabolic biomarkers related to Cd exposure after 24, 48 and 96 h. Metabolite assignments in the 1-dimensional 500-MHz ^1^H NMR spectra of tissue extracts from the gills of *T*. *granosa* are shown in Supplementary Table S1. After Cd exposure for 24 h, the gill samples from the control and both doses (25 and 250 μg/L) of Cd-exposed groups were clearly separated in OPLS-DA score plots (Fig. 2A,C), with reliable *Q*^2^ values greater than 0.8. Based on the loading plot of OPLS-DA (Fig. 2B,D), both doses (25 and 250 μg/L) of Cd induced identical metabolic responses in blood clam gill tissues. Although the Cd concentration differed by 10 times in water between the 2 exposure groups, the difference was 1.9 times in tissues between them. This result could explain the identical metabolic responses that were induced by both doses of Cd in blood clam gills at 24 hpe. For those blood clam samples that were sampled at 48 hpe (Supplementary Fig. S2), the low dose of Cd (25 μg/L) increased the levels of alanine, glutamine and succinate and decreased the levels of leucine, aspartate, adenine and proline. Although the high dose of Cd (250 μg/L) induced some similar metabolic responses, including increased alanine, glutamine and succinate and decreased leucine, aspartate and proline, glycogen was uniquely elevated, and adenine was not altered in this dose of Cd-exposed blood clam gill tissues. For those blood clam samples that were sampled at 96 hpe (Supplementary Fig. S3), the results indicate that the low dose of Cd caused increases in alanine, glutamate, succinate and glycogen and decreases in leucine, β-alanine, hypotaurine, proline and adenine, whereas the high dose of Cd induced similar metabolic biomarkers, including increased alanine, glutamate, succinate and glycogen and decreased leucine, β-alanine, hypotaurine and proline.

Overall, the dose- and time-dependent toxicological effects of Cd exposure included neurotoxicity and disturbances in energy metabolism after exposure for 24 and 48 hours, as indicated by elevated glutamate, glutamine, alanine, succinate and decreased leucine, aspartate and proline^20^,^33^. After exposure for 96 hours, however, both Cd exposures induced disturbance in the osmotic regulation in blood clam gill tissues, as displayed by decreased hypotaurine and β-alanine. Interestingly, both Cd exposures induced identical metabolic biomarkers at 24 h post-treatment and no significant difference between the two doses (25 and 250 μg/L) of Cd-exposed groups. This result probably can be explained by the similar levels of accumulated Cd in blood clam gill tissues. Thus, we selected the high dose 250 μg/L Cd-exposed group for further proteomic analysis.

### GO and KEGG analysis of the differential expression proteins at three time points

**Figure 3:**
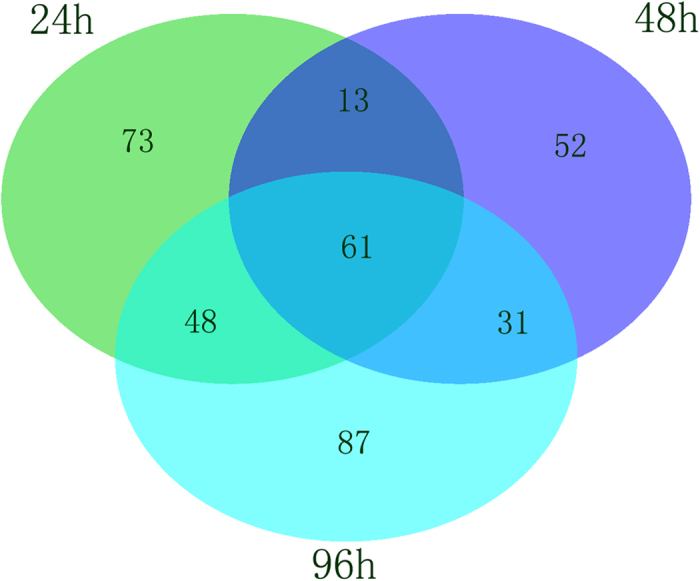
Changed proteome distribution between different time points. Venn diagram showing unique and shared proteins between time points.

**Figure 4:**
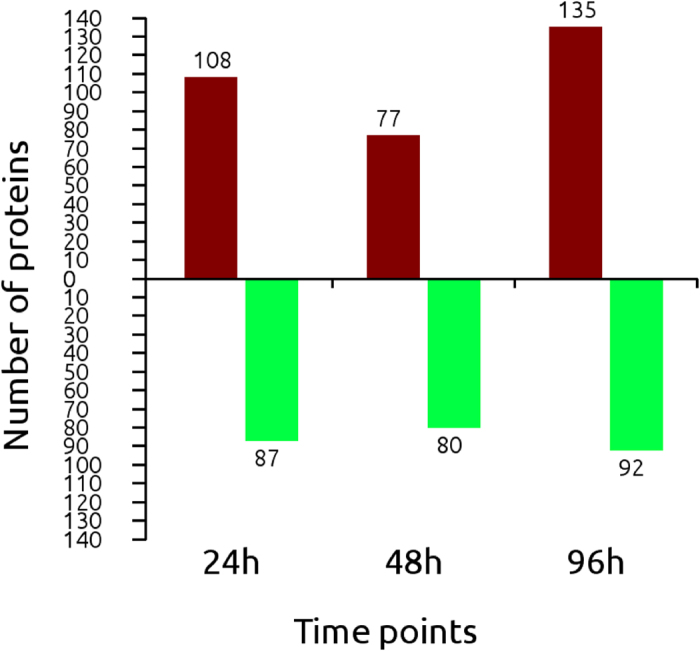
Number of differential expressed proteins detected at 24, 48 and 96 h.

Only proteins that contained at least two peptides and that were detected in both replicates were used for quantification; we described global proteome changes in the *T*. *granosa* gill during the short-term course of Cd exposure. We identified 2329 distinct proteins, of which 2318 were identified and quantified reliably at a global false discovery rate (FDR) of 1%. 365 identified proteins had significant changes in expression at different time points, compared with the control group, and 61 proteins were differentially expressed at all of the examined time points (Fig. 3, Supplementary Table S2). Among these differentially expressed proteins, 195, 157 and 227 up- or down-regulated proteins were identified in Cd-challenged *T*. *granosa* at 24, 48 and 96 h, respectively (Fig. 4).

**Figure 5:**
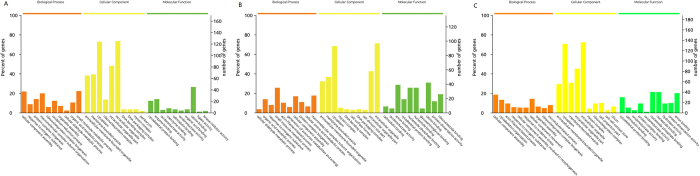
Most significant enriched GO terms of differentially expressed proteins at each time point. (**A**) 24 h; (**B**) 48 h; and (**C**) 96 h.

**Figure 6:**
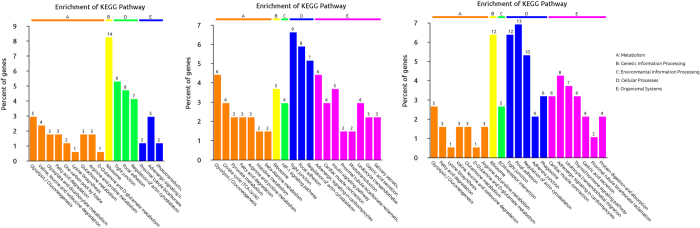
Most significant associated KEGG pathways of differentially expressed proteins at each time point. (**A**) 24 h; (**B**) 48 h; and (**C**) 96 h.

For an overview of the function of all of the detected differential expression proteins (DEPs) and the potential linkage between them, a GO enrichment analysis was performed for DEPs detected at each time point. The DEPs were classified into the Biological process (BP), Cellular component (CC) and Molecular function (MF) categories. The DEPs were predominantly binding proteins that were involved in cellular component organization or biogenesis and in the response to inorganic substances 24 h post Cd treatment. At 48 hpe, DEPs were also primarily binding proteins that were involved in the cellular aldehyde metabolic and organic acid metabolic processes, and at 96 hpe, DEPs were predominantly proteins that were related to cellular component assembly and cytoskeleton organization (Fig. 5). Further KEGG pathway enrichment revealed that these proteins were mainly involved in glycolysis/gluconeogenesis, ribosomes, tight junctions and proximal tubule bicarbonate reclamation at 24 hpe; glycolysis/gluconeogenesis and the HIF-1 signaling pathway at 48 hpe; and glycolysis/gluconeogenesis, ribosomes, ECM-receptor interaction and tight junctions at 96 hpe (Fig. 6). The common pathways from three time points are glycolysis/gluconeogenesis, tight junctions and ribosomes, which should be closely related to Cd toxicity and metabolism.

In humans, Cd induces changes in carbohydrate metabolism and gluconeogenesis through various effects on the liver, kidneys, adrenal glands and pancreas. Laboratory-controlled injection and ingestive intake of Cd produce an increase in gluconeogenesis and blood glucose^34^. Cadmium induced gluconeogenic changes in rat kidney and liver have been reported^35^. Tight junctions mediate cell-cell adhesion and can regulate protein transport through the extracellular matrix^36^. *In vivo*, disrupted tight-junction associated microfilaments in rat sertoli cells were detected after Cd exposure^37^. It suggests that tight junction, i.e., intracellular adhesion and the interaction of the actomyosin cytoskeleton with the plasma membrane were likely associated with Cd in blood clams^38^. It is likely that Cd in blood clams could destroy the function of tight junctions, i.e., intracellular adhesion and the interaction of the actomyosin cytoskeleton with the plasma membrane^38^. Ribosomal genes have been reported to be early targets of cadmium-induced toxicity in *Chironomus riparius* larvae, indicating that cadmium can interfere cellular survival via interaction with ribosomal genes that led to extreme impairment of nucleolus activity^39^. Significant quantities of cadmium have been found in purified ribosomes and from rat liver, people have detected significant quantities of Cd, also suggesting the attachment of Cd with ribosomes^40^.

### GO and KEGG analysis of common DEPs of three time points

Among all DEPs, 61 proteins were detected in all three time points. A GO and KEGG analysis of 61 common DEPs showed that the functions of its DEPs are distributed in cytoskeletal protein binding, calmodulin binding and oxidoreductase activity, which are involved in the glycolysis/gluconeogenesis, tight junction and adrenergic signaling pathways (Supplementary Figs S4 and S5).

Moreover, altered glycolysis was also seen in our NMR-based metabolomics study, with four metabolites, glutamine, alanine, glucose and glycogen, which related to glycolysis/gluconeogenesis process^41^, all up-regulated after Cd exposure. The results of proteomics and metabolomics coincide, showing that up-regulation of glycolysis/gluconeogenesis pathways are important for Cd metabolism in *T*. *granosa*. Similar responses were found in plant tomato roots after Cd treatment^42^. The breakdown of tight junction is a sign of cadmium treatment reported in previous studies^37^,^43^, and our results also indicate that cadmium affects the integrity of tight junctions in blood clam.

### Proteome characterization induced by Cd exposure

Some well-documented stress- or immunity-related, calcium-binding and sulfur-compound metabolism proteins were found with large fold-changes at three time points (Table 1). The universal stress protein A-like protein was significantly up-regulated at all three time points and sharply increased to 24.88-fold compared to that of the control group after 96 h of exposure. The universal stress proteins (USPs) appear to play an active role in the abiotic stress response in bacteria and plants^44^,^45^, but their functions remain largely unknown in animals. Cu/Zn superoxide dismutase was also highly expressed, increasing by more than 10-fold compared to that in the control at all three time points. It is generally agreed that oxidative stress plays important roles in acute Cd poisoning^46^; Cd-generated superoxide anion, hydrogen peroxide, and hydroxyl radicals have been detected *in vivo* by electron spin resonance spectra^47^. Cu/ZnSOD is an important antioxidant enzyme that catalyzes the conversion of superoxide to oxygen and hydrogen peroxide in aerobic organisms. The study also shows that the metal-response element (MRE) in the proximal 5’ flanking sequence of Cu/ZnSOD is required for the induction of the gene by heavy metals^48^. Therefore, it is very important that Cu/ZnSOD contributes to detoxifying Cd poisoning and maintaining the dynamic balance of ROS. Increasing evidence suggests that the toxicity of Cd is mediated by oxidative stress-induced cell death. The important antioxidant defense enzyme glutathione S-transferase (GST) was also up-regulated; other studies have reported that GST plays a critical role in the detoxification of Cd^49^. Cd can disrupt Ca^2+^ homeostasis, causing apoptosis in a variety of cells^50^. In the present study, we found that some Ca^2+^ related proteins were significantly up-regulated after Cd exposure. Three proteins, including neuronal calcium sensor (Ncs1), sarcoplasmic calcium-binding protein (Hrc) and calmodulin (Calm1), were up-regulated more than 5-fold compared with the control group. The biophysicochemical similarities of Cd^2+^ and Ca^2+^ allow Cd^2+^ to displace Ca^2+^ in some Ca^2+^-binding proteins and to disrupt Ca-mediated signaling pathways^51^. Further determination of the short-term and long-term factors that are affected by spatial and molecular interactions of Cd^2+^ and Ca^2+^ will provide additional insight into toxic mechanisms.

In plants, many studies have shown that Cd can induce sulfate uptake and that sulfur metabolism is associated with the detoxification of Cd^52^,^53^,^54^,^55^,^56^. However, few studies have reported the Cd and sulfur compound relationship in animals. In our study, we found that sulfotransferase (Sult1b1) was significantly up-regulated after Cd challenge. This result suggests that sulfur-related metabolism might also be involved in the Cd-induced response and the detoxification of Cd in animals.

Heavy metals can change nucleosome structure by binding to its core component and this may have a potential function in toxicity^57^. In our study, the key nucleosome components H2, H3 and H4 significantly decreased after Cd exposure. It might implicate Cd-induced DNA damage and apoptosis^58^. Another study showed that heavy metal exposure results truncation of H2A and H2B, and the elimination of some functional modification sites^59^. Similar metabolism progress might also appear in the cell after Cd treatment, probably resulting in the truncation of histones.

### Regulation model construction

**Figure 7:**
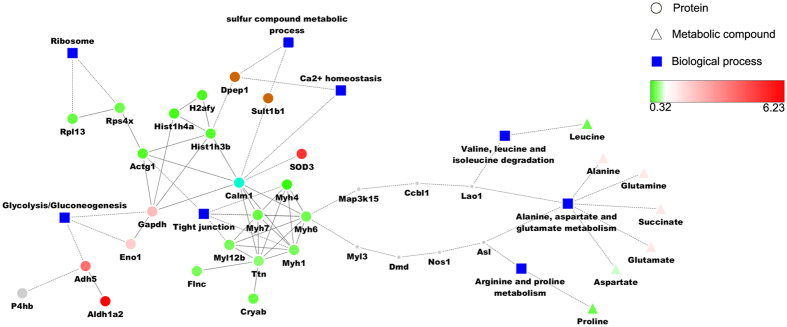
Key regulation model constructed using proteomic and metabolomic data. Circle nodes indicate proteins and triangle nodes indicate metabolic compounds. Filled color represents expression fold change using gradient color from blue to red. Blue colored rectangle indicates KEGG pathways. A solid line between two proteins indicates a known interaction annotated in the STRING database; a dashed line between proteins indicates indirect interaction. Gray-colored dots are extended proteins from the database that built up a potential path from the proteomic to the metabolomic layer.

A regulation network was constructed to better understand the relationship between the proteins, affected pathways and downstream metabolic compounds. As shown in the model (Fig. 7), Rpl13 and Rps4x, two ribosome proteins, were significantly down-regulated at all three time points; the expression of the glycolysis-related proteins, Adh5, Eno1 and Gapdh notably increased, suggesting an activation of glycolysis in Cd stimulation; and Myh7, Actg1, Myh6, Myl12b, Myh4, and Myh1, which are involved in tight junctions, were suppressed. Histone proteins, such as H2afy, Hist1h3b and Hist1h4a were also markedly down-regulated; these findings are consistent with those of previous studies^58^. Moreover, the model demonstrates a potential crosstalk between these pathways: protein Actg1 (cytoplasmic actin) is a connector of ribosomes, histones and tight junctions; and Gapdh (glyceraldehyde-3-phosphate dehydrogenase) is overexpressed, linking glycolysis and histones together. Gapdh also interacts with Actg1, suggesting the probable inhibitory function of Gapdh in the Cd response. Calm1, a Calmodulin, mediates the control of a large number of Ca^2+^-driven enzymes, kinases and phosphatases involved in cytokinesis; it is a connector of tight junctions and histones. Interestingly, Calm1 was first overexpressed by more than 1.9-fold at 24 h and then inhibited at 48 h and 96 h. Calm4 (not shown in the model), another calcium-binding protein, shows a similar profile as that of Calm1, first being overexpressed by 2.2-fold at 24 h and 48 h and then down-regulated at 96 h. These results suggest that the level of calcium-binding proteins is crucial in protection against Cd. Associated with Calm1 is SOD3 (extracellular Cu/ZnSOD), an important anti-toxic protein protecting the extracellular space in biological systems. The expression of this protein increased more than 10-fold at all 3 time points, and the expression of SOD1 (not shown in model) increased more than 10-fold only at 96 h, suggesting that SOD1 might function as a backup enhancer that activates under long-term Cd exposure. The complementary expression profile of SOD1 and Calm4, SOD3 and Calm1 might suggest vital novel regulation signaling controlled by the SOD and Calm family.

In tight junctions, members of the Myosin-II family are inhibited by Cd, including Myh1, 4, 6, 7 and 12b^60^; these proteins are key components in the assembly and contraction of muscle^61^. The model also simulates a connection possibility of the key differentially expressed proteins with significantly-expressed metabolic amino acids. This model shows 2 possible paths linking amino acid metabolism pathways, including valine, leucine and isoleucine degradation; alanine, aspartate and glutamate metabolism; and arginine and proline metabolism. One possible path is associated with map3k15 (mitogen-activated protein kinase kinase kinase 15 Gene), ccbl1 (cysteine conjugate-beta lyase 1), and Lao1 (L-amino acid oxidate 1); these proteins might be involved in oxidative stress processes^62^,^63^. The other pathway is through myl3 (myosin, light polypeptide 3), Dmd (Dystrophin), Nos1 (nitric oxide synthase 1), and Asl (argininosuccinate lyase), an important player in the arginine and proline metabolic pathway, as indicated by the KEGG database. This pathway might be related to NO regulation, a messenger of neurotransmitter signals in the nervous system^64^,^65^,^66^,^67^.

In summary, the model illustrates a possible regulation network consisting of important proteome and metabolomic changes upon Cd stimulation. It suggests that the ribosome was likely down-regulated, glycolysis was likely activated and tight junctions were likely suppressed. These pathways were interlinked with down-regulated histone complex. Compromising tight junctions is likely to interfere with oxidative stress and NO regulation, and in turn be responsible for up-regulated calcium-binding related proteins and downstream up-regulated amino acid metabolism. Noteworthy, some previous studies have reported response of Cd in evolutionally distant species rather than clam^20^,^51^. Although we cannot directly compare the changes at molecular level, genes in clam do not necessarily have homologous in distant related species, and Cd does not necessarily effect same genes in different species. But we have found relationship at the biological process level, such as tight junction system and glycolysis system of both tomato and clam were significantly affected by Cd exposure. This study provides a potential regulation network model consistent of these biological processes based on mammalian interactomes, further investigations should be done to validate the key molecules and interactions.

## Conclusion

Cd toxicity is a multigenetic, complex process. The use of proteomics and metabolomics has provided insight into the comprehensive landscape of change at the molecular level. A total of 365 proteins and 12 metabolites were significantly changed after Cd exposure. Interestingly, the metabolic biomarkers were consistent in the time series analysis, suggesting a conserved mechanism of Cd toxicity. To explore the core mechanism, we focused on differentially expressed proteins, independent of the exposure time. Bioinformatic analysis identified a core connected regulatory network consisting of 15 down-regulated proteins; these proteins were functionally involved in histone function, ribosomal process and tight junctions. This result might suggest that histones and ribosomes are the primary targets of Cd. Glycolysis, sulfur metabolic process and Ca^2+^ homeostasis were also detected in our study, and these processes were connected with the core network, suggesting the mechanisms of Cd toxicity were interlinked. In contrast, the myosin complex was significantly suppressed, leading to the abnormal function of the tight junction, which could be a potential mechanism to disturb the neural and osmotic function of tissue. This work is the first multi-omics study of the basic mechanism of Cd toxicity and the results provide a list of potential key regulators of Cd toxicity in blood clam *T*. *granosa*. The continuous functional investigation of these proteins could eventually solve the key targets of Cd toxicity.

## Methods

### Clam, exposure conditions, and sample collection

Adult blood clams *T*. *granosa* (shell length: 2.8–3.2 cm) were collected from a local culturing farm in Ningbo, China. These clams were allowed to acclimate in aerated seawater (20 °C, 30‰ salinity, collected from a pristine environment) in the laboratory for 2 weeks and fed with *Platymonas subcordiformis* at 2% of the tissue dry weight per day. After acclimatization, all the blood clams were divided into three 50-L tanks (three cadmium exposure levels 0, 25, 250 μg/L), each containing sixty blood clams. For Cd exposure, the blood clams were exposed to dissolved 25 and 250 μg/L Cd^2+^, respectively. Cadmium solution (12.5 g/L) were prepared from Cd(NO_3_)_2_·4H_2_O (analytical grade), 0.1 L and 1 L cadmium solution were added to 49.9 L and 49 L sea water for final concentrations of 25 and 250 μg/L Cd^2+^, respectively. The low experimental concentration (25 μg/L) of Cd^2+^ can be found in heavily polluted seawaters along the coast of China^51^. At each sampling time point (24, 48 and 96 hpe), fifteen blood clams from the control and Cd-exposed groups were immediately dissected for the gill tissues (~100 mg). In total, 180 clams representing five biological replicates and four time points (0 h as control) of three experiments were used for the following cadmium concentration determination, proteomic and metabolomics analysis. All of the gill tissues were flash frozen in liquid nitrogen and then stored at −80 °C.

### Cadmium concentration determination

Five gill samples were dried and digested according to Wu *et al*. lab protocol^20^. PE Analyst-800 was used to quantify the Cd concentration. For internal quality control, we have used the tissue of GBW08571 marine muscle as certified reference^68^. Three individual spiking experiments were performed to recovery the target elements. Restriction level for Cd was set to 95.6–104.3%. One-way ANOVA followed by an unpaired student t-test was applied. *P*-value  < 0.05 was considered as significant. SPSS19 software was used for the statistical analysis. The blood clams (*T*. *granosa*) here are commercially cultured animals, and all the experiments were conducted in accordance with the recommendations in the Guide for the Care and Use of Laboratory Animals of the National Institutes of Health. The study protocol was approved by the Experimental Animal Ethics Committee of Zhejiang Wanli University, China.

### Metabolomics analysis

Modified extraction protocol^69^ was used to obtain polar metabolites from gills. Briefly, 100 mg gill tissue was homogenized (Percellys 24, Bertin Technologies, France) and mixed with 4 mL/g methanol, 0.85 mL/g water, and 2 mL/g chloroform, then shaken and centrifuged at 4 °C, 3000 g, for 5 minues. The supernatant substance was then mixed with 2 mL/g chloroform and 2 mL/g water, vortexed and centrifuged for 10 minutes, at 3000 g, 4 °C. The methanol/water layer was dried, re-suspended in 600 μL of 100 mM phosphate buffer (Na_2_HPO_4_ and NaH_2_PO_4_ with 0.5 mM TSP (sodium 3-trimethylsilyl [2, 2, 3, 3-^2^D_4_] propionate) as the internal standard for the chemical shift, pH 7.0) in D_2_O, vortexed and centrifuged for 5 minutes at 3000 g, 4 °C. The supernatant substance was used for NMR analysis.

One-dimensional ^1^H NMR spectra of gill extracts were acquired on a Bruker AV 500 NMR spectrometer performing at 500.13 MHz at 298 K^30^. All the ^1^H NMR spectra were acquired using water suppressed NOESYPR1D pulse sequence (recycle delay-90°-*t*_1_-90°-*t*_m_-90°-acquisition). Sixteen K data points of the NMR signal were acquired with a 6009.6 Hz spectral width, an acquisition time of 2.7 s, and a recycle delay time of 3.0 s using 128 scans. The water suppression was achieved with the selective irradiation on the water peak during a fixed interval t_1_ of 4 μs and the mixing time t_m_ of 100 ms. Then, the datasets were zero-filled to 32,768 points, and exponential line-broadenings of 0.3 Hz were applied before Fourier transformation. All ^1^H NMR spectra were phase- and baseline corrected and chemical shift was calibrated to TSP (δ 0.00) manually using TopSpin (version 2.1, Bruker). Then, each spectrum was segmented into 0.005 ppm bins between 0.2 and 9.1 ppm with bins from 4.72 to 4.96 ppm (water) excluded from all the NMR spectra. The area for each segmented region was calculated and normalized to the total integrated area of the spectra. All the NMR spectra were generalized by log transformation to stabilize the variance across the spectral bins and to increase the weightings of the less intense peaks.

Partial least squares discriminant analysis (PLS-DA) and orthogonal projection to latent structure with discriminant analysis (OPLS-DA) were performed on the NMR data of clam groups at 24 h, 48 h, 96 h against at 0 h, respectively^70^. The quality of the constructed PLS-DA models was assessed using cross-validation with five-way split Venetian blinds. A Q^2^ score of >0.08 indicates that the PLS-DA model is significantly better than chance, while a score between 0.7 and 1.0 indicates that the model is highly robust and the corresponding Latent Variable weight plots of OPLS-DA can usually present the contributive NMR bins (peaks) of metabolites for the separation between control and exposed groups^71^. The validity of each constructed OPLS-DA model was evaluated with the cross validation-ANOVA approach (*p* < 0.05). The OPLS-DA results were visualized in score plots to show the classifications and in corresponding loading plots to show the NMR spectral variables contributing to the classifications. Score plots were used for classification visualization and corresponding loading plots were used to show variables contribution. Then, MATLAB (V7.0, MathWorks Inc., Natwick, USA) was used to perform coefficient-coded loadings plots with an in-house-developed program and were color-coded with the absolute value of the correlation coefficients (r). The metabolites with absolute value of r above 0.602 were considered to be statistically significant (*p* < 0.05). Chenomx (Evaluation Version, Chenomx Inc., Edmonton, Alberta, Canada) was used for metabolites assignments.

### Proteomics analysis

Five biological replicates were mixed, then divided into two experimental replicates of each time point of the 250 μg/L Cd^2+^ group were prepared. Total protein of each gill sample was powdered with liquid nitrogen. Mixture of the powder and 9 M urea were centrifuged at 15,000 g for 15 min at 4 °C. Bradford method was used for supernatant quantification^72^. The samples were then centrifuged at 15,000 g for 15 min at 4 °C. The supernatant was collected and quantified by the Bradford method. After applying 20-μg samples to a 12% SDS-PAGE gel, the gel was visualized by CBB stain according Candiano’s protocol for checking the protein^73^.

100 mg of protein for each sample were mixed with a dissolution buffer of AB sciex (Foster City, CA, USA), digested with trypsin and labeled with iTRAQ reagents 8-plex kit (AB sciex). Samples and independent biological replicates were labeld 113–117,114–118,115–119, and 116–121 Da, respectively. Labeled samples were then pooled and dried in an Eppendorf vacuum concentrator.

Agilent 1200 HPLC was used for sample purify, Exigent nanoLC-Ultra 2D system (AB SCIEX) was used for sample separation. Triple TOF 5600 mass spectrometer and a Nanospray III source (AB SCIEX) was used to perform Mass spectrometer data acquisition.

Protein Pilot (v4.5) was used for raw data processing against the *T*. *granosa* transcription and *C*. *gigas* genome database^74^. Protein identification was performed with the search option of emphasis on biological modifications. The database search parameters were as follows: the instrument was TripleTOF 5600, iTRAQ quantification, cysteine modified with iodoacetamide; and biological modifications were selected as ID focus and trypsin digestion. Peptides with global false discovery rate (FDR) < 1% were used for further protein annotation. Average ration of 114/118, 113/117 at 24 h, 115/119 and 113/117 at 48 h, and 116/121 and 113/117 at 96 h were assigned as fold change value. Proteins with a corrected fold change of >1.50 or <0.67 in any duplicate were considered to be significantly differentially expressed.

### Bioinformatics Analysis

Blastp was used against the *Mus musculus* proteome database at uniprot (http://www.uniprot.org/). The Protein with best identity to query protein among the best 10 hits with e-value <1e^−10^ was considered as homologous of the gill proteins in mouse. DAVID toolkit was used for gene ontology enrichment analysis. Associated KEGG signaling pathways were calculated using fish’s exact tests. The STRING database (version 9.1)^75^ was used to obtain protein-protein interaction information with default score setting. Potential paths of differentially expressed proteins and metabolic compounds were searched in the knowledge-driven database with a shortest-path algorithm. Cytoscape (http://cytoscape.org/) version 3.1 was used for regulation network construction.

## Additional Information

**How to cite this article**: Bao, Y. *et al*. Identification of a regulation network in response to cadmium toxicity using blood clam *Tegillarca granosa* as model. *Sci. Rep.* **6**, 35704; doi: 10.1038/srep35704 (2016).

## Supplementary Material

Supplementary Information

## Figures and Tables

**Table 1:** Summary of differential expressed protein after 96 h Cd exposure (fold change >2 or <0.5 in all three time points).

Biological function	Protein description	E-value	Protein accession no.	Fold change		
				24 h	48 h	96 h
Stress response	universal stress protein A-like protein	1e-53	XP_011429497	17.39	4.09	24.88
	core histone macro-H2A	0.0	XP_011454400	0.19	0.39	0.33
	histone H2A isoform 2	3e-79	ACJ12611	0.22	0.39	0.53
Antioxidant defense	Cu/Zn superoxide dismutase	4e-40	AFQ32467	10.29	10.43	11.81
	glutathione S-transferase	6e-83	AGN03945	—	1.90	3.16
Calcium-binding protein	neuronal calcium sensor (EF-hand motif)	4e-05	P36608	6.36	11.47	2.20
	sarcoplasmic calcium-binding protein	2e-80	BAA89417	5.43	5.30	3.27
	calmodulin	4e-18	XP_010966045	6.36	11.47	2.20
Sulfur compounds metabolism	sulfotransferase	4e-92	XP_004633325	6.39	7.40	5.06
	protein disulfide-isomerase	8e-49	ERE68284	—	2.03	2.72
Cell structure	tenascin-X	0.24	XP_006537513	3.95	4.23	3.00
	mucin-17-like	2e-136	XP_011425282	3.25	4.12	4.91
	tropomyosin	3e-28	XP_011437747	3.20	4.14	3.41
Metabolic process	retinal dehydrogenase	0.0	XP_006743256	4.00	6.23	4.29
	isocitrate dehydrogenase	0.0	EKC40376	3.37	2.08	3.05
	phosphohistidine phosphatase	8e-47	XP_004848882	3.14	2.93	2.55
	annexin	5e-137	XP_004561320	3.07	2.96	5.47
	protein mab-21	9e-58	XP_004549366	3.00	4.62	3.66
	1,4-alpha-glucan-branching enzyme-like	0.0	XP_011434285	2.85	3.20	3.30
	glyceraldehyde-3-phosphate dehydrogenase	0.0	XP_010609934	2.51	2.45	3.06
	hemoglobin I	3e-37	AGA03854	2.45	2.23	2.21
	thioredoxin	6e-33	XP_002504076	2.41	4.63	3.85
	dipeptidase 1-like	3e-110	XP_011438699	2.31	2.38	2.51
	enolase-like	5e-115	XP_011436227	2.10	2.05	2.33
	pyruvate carboxylase	0.0	EKC30574	2.05	2.89	2.22
	complement C1q tumor necrosis factor-related protein	2e-14	XP_011436150	0.09	0.43	0.28
	collagen alpha-1	1e-08	XP_006792865	0.21	0.32	0.29
