# Supplementary material for: Identification of a regulation network in response to cadmium toxicity using blood clam Tegillarca granosa as model
Source: Sci Rep. 2016 Oct 20;6:35704. doi: 10.1038/srep35704 (PMC5071765; doi:10.1038/srep35704)
Supplement: Supplementary Information [file srep35704-s1.pdf]

Identification of a regulation network in response to cadmium toxicity using blood clam *Tegillarca granosa* as model

Yongbo Bao[1], Xiao Liu[3], Weiwei Zhang[2], Jianping Cao[4], Wei Li[1], Chenghua Li[2†], Zhihua Lin[1†]

[1]Zhejiang Key Laboratory of Aquatic Germplasm Resources, College of Biological & Environmental Sciences, Zhejiang Wanli University, Ningbo, Zhejiang, 315100, PR China. [2] School of Marine Scienes, Ningbo University, Ningbo, Zhejiang, 315010, PR China. [3]Department of Systems biology, GFK, Shanghai Biotech Inc, Shanghai, 201112, PR China. [4]Ningbo Yinzhou Measurement and Test Center for Quality and Technique Supervising, Ningbo, Zhejiang, 315100, PR China.

[†]Address correspondence to Zhihua Lin, zhihua9988@126.com

Supplementary Figures S1 to S5

Supplementary Table S1 and S2

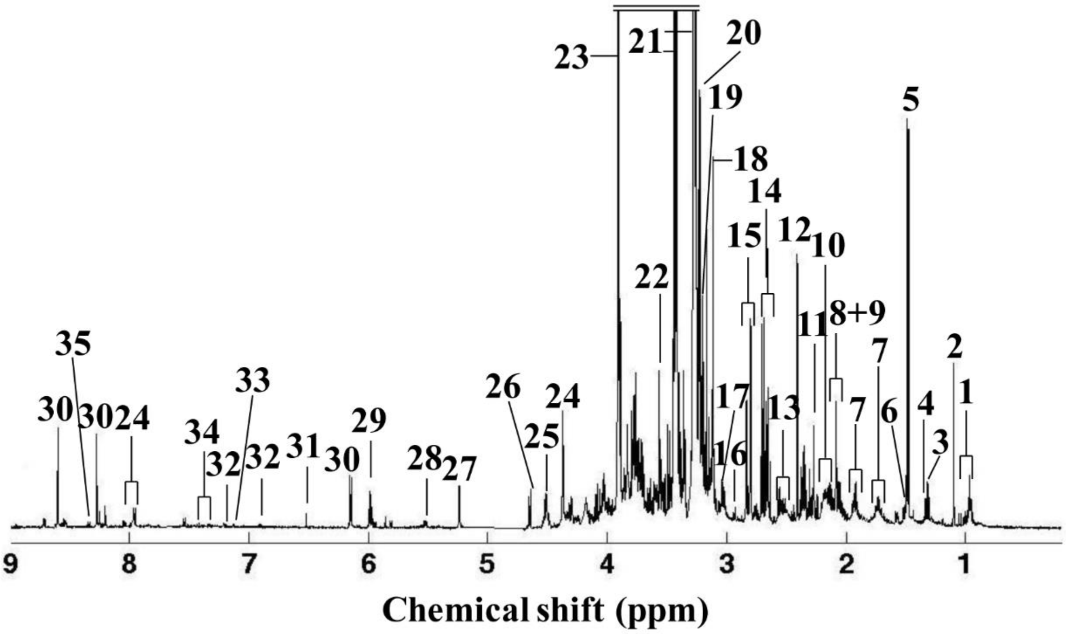

Supplementary Figure 1 | A representative 1-dimensional 500 MHz ^{1}H NMR spectrum of gill tissue extracts from one control blood clam *Tegillarca granosa*. **Keys:** (1) branched chain amino acids: leucine, isoleucine and valine, (2) unknown 1 (1.10 ppm), (3) threonine, (4) lactate, (5) alanine, (6) unknown 2 (1.51 ppm), (7) arginine, (8) proline, (9) glutamate, (10) glutamine, (11) acetoacetate, (12) succinate, (13) β-alanine, (14) hypotaurine, (15) aspartate, (16) asparagine, (17) lysine, (18) malonate, (19) choline, (20) phosphocholine, (21)taurine, (22) glycine, (23) betaine, (24) homarine, (25) AMP, (26) β-glucose, (27) α-glucose, (28) glycogen, (29) unknown 3 (5.95 ppm), (30)ATP, (31) fumarate, (32) tyrosine, (33) histidine, (34) phenylalanine (35) adenine.

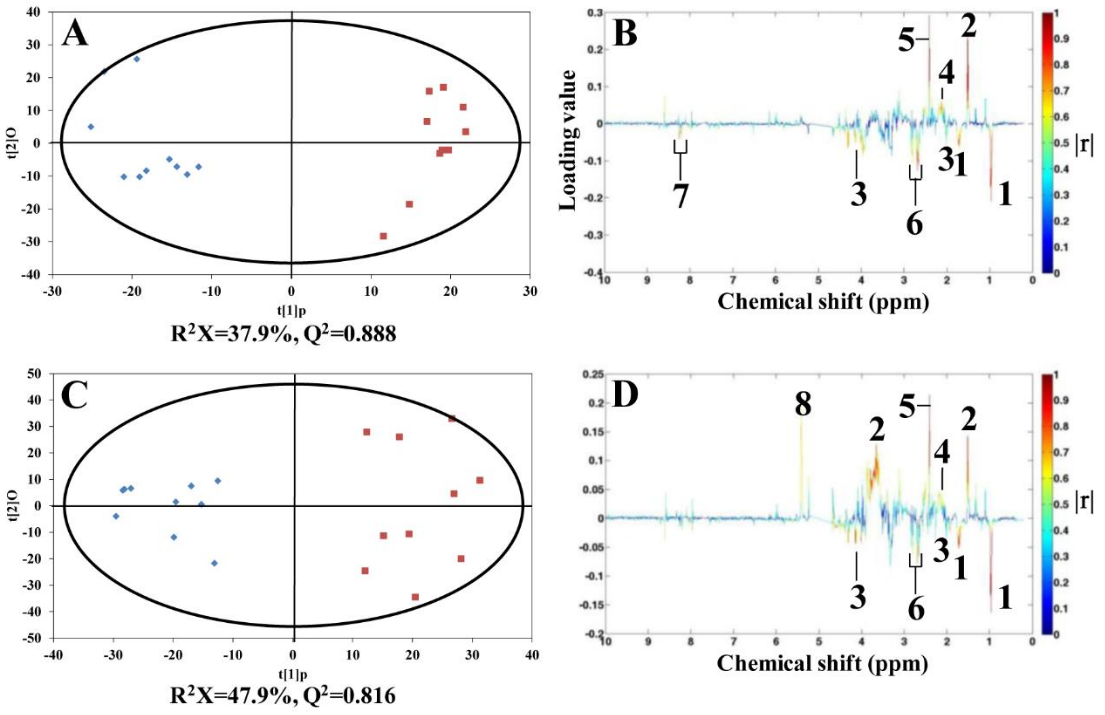

Supplementary Figure 2 | O-PLS-DA score plots derived from the ^{1}H NMR spectra of gill tissue extracts from the control (♦) and exposed (■) blood clam groups with (A) 25 and (C) 250 μg/L Cd, respectively, after exposure for 48 hours and corresponding coefficient plots (B) and (D). **Key:** (1) leucine, (2) alanine, (3) proline, (4) glutamine, (5) succinate, (6) aspartate, (7) adenine and (8) glycogen.

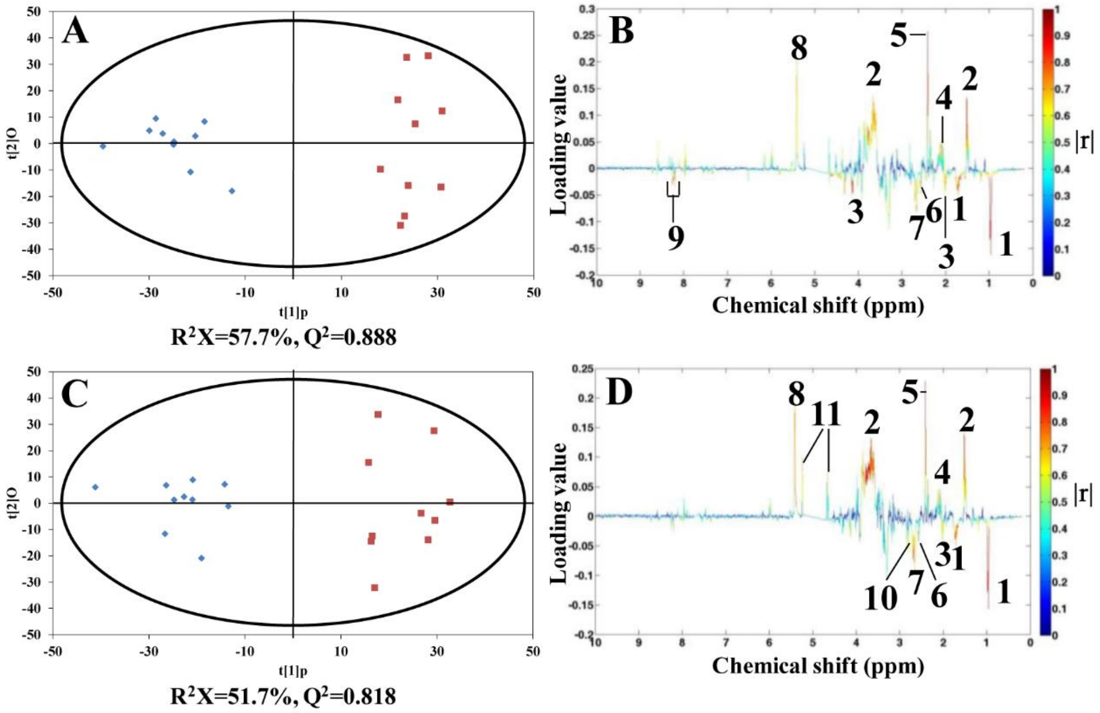

Supplementary Figure 3 | O-PLS-DA scores plots derived from ^{1}H NMR spectra of gill tissue extracts from the control (♦) and exposed (■) blood clam groups with (A) 25 and (C) 250 µg/L Cd, respectively, after exposure for 96 hours and corresponding coefficient plots (B) and (D). **Key:** (1) leucine, (2) alanine, (3) proline, (4) glutamate, (5) succinate, (6) β-alanine, (7) hypotaurine, (8) glycogen, (9) adenine, (10) aspartate and (11) glucose.

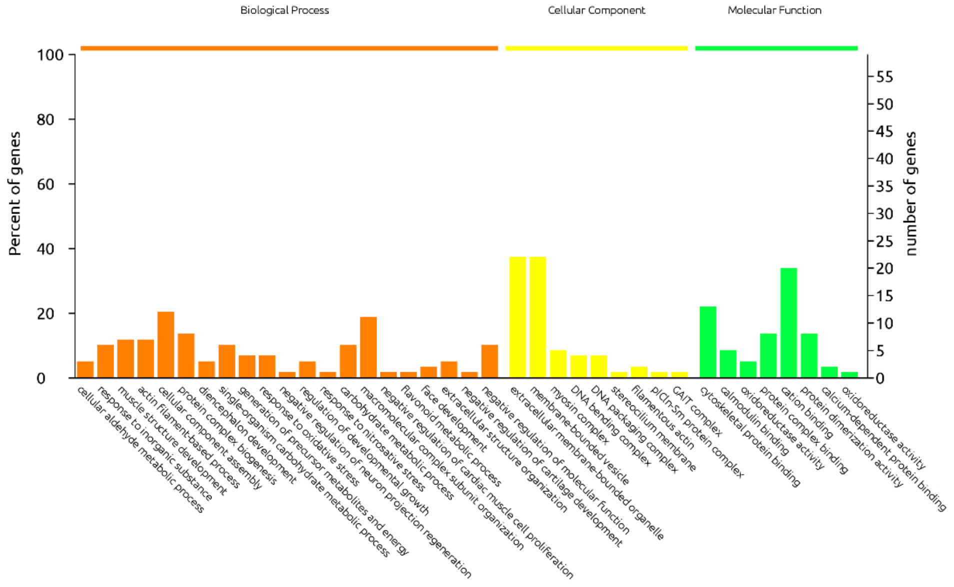

Supplementary Figure 4 | Go enrichment analysis of 61 common DEPs of three time points.

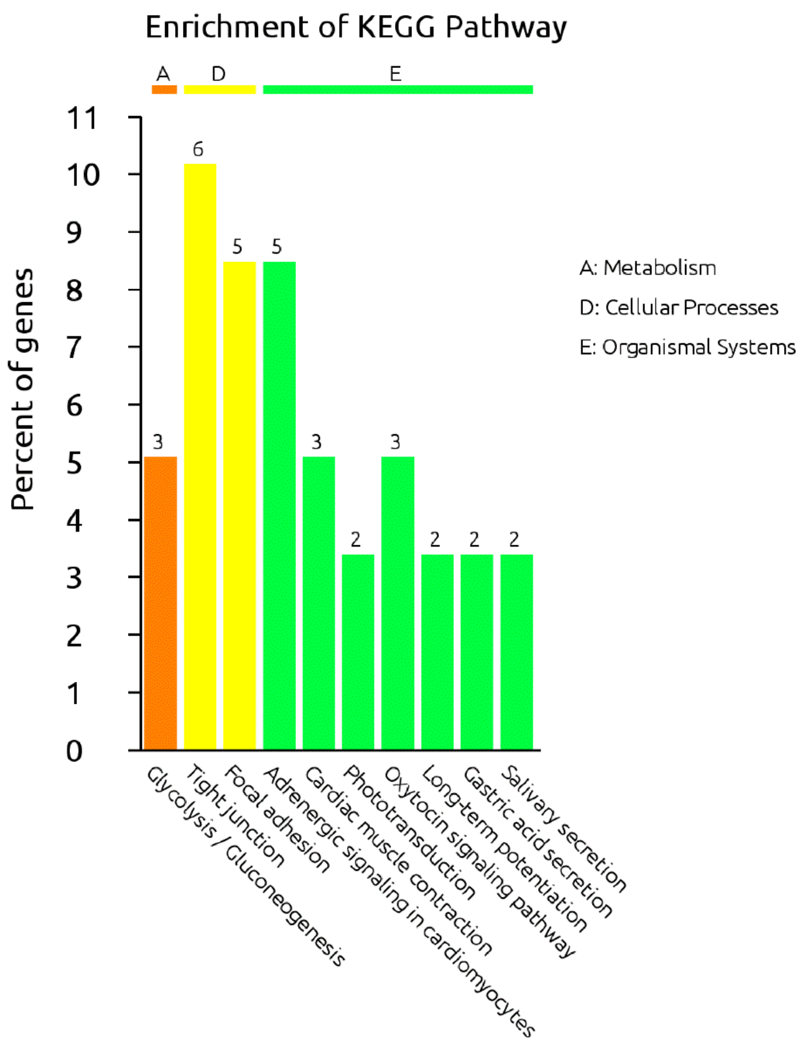

Supplementary Figure 5 | KEGG pathway enrichment analysis of 61 common DEPs of three time points.
